# Metal Oxide Heterostructures for Improving Gas Sensing Properties: A Review

**DOI:** 10.3390/ma16010263

**Published:** 2022-12-27

**Authors:** Fan-Jian Meng, Rui-Feng Xin, Shan-Xin Li

**Affiliations:** 1 State Key Laboratory of Advanced Metallurgy, School of Metallurgical and Ecological Engineering, University of Science and Technology Beijing, Beijing 100083, China; 2 School of Materials, Sun Yat-sen University, Shenzhen 518107, China

**Keywords:** metal oxide semiconductor, sensing materials, heterostructures, synthesis methods, gas sensing mechanism

## Abstract

Metal oxide semiconductor gas sensors are widely used to detect toxic and inflammable gases in industrial production and daily life. The main research hotspot in this field is the synthesis of gas sensing materials. Previous studies have shown that incorporating two or more metal oxides to form a heterojunction interface can exhibit superior gas sensing performance in response and selectivity compared with single phase. This review focuses on mainly the synthesis methods and gas sensing mechanisms of metal oxide heterostructures. A significant number of heterostructures with different morphologies and shapes have been fabricated, which exhibit specific sensing performance toward a specific target gas. Among these synthesis methods, the hydrothermal method is noteworthy due to the fabrication of diverse structures, such as nanorod-like, nanoflower-like, and hollow sphere structures with enhanced sensing properties. In addition, it should be noted that the combination of different synthesis methods is also an efficient way to obtain metal oxide heterostructures with novel morphologies. Despite advanced methods in the metal oxide semiconductors and nanotechnology field, there are still some new issues which deserve further investigation, such as long-term chemical stability of sensing materials, reproducibility of the fabrication process, and selectivity toward homogeneous gases. Moreover, the gas sensing mechanism of metal oxide heterostructures is controversial. It should be clarified so as to further integrate laboratory theory research with practical exploitation.

## 1. Introduction

As people pay increasingly more attention to the environmental protection, the detection of toxic, inflammable, and explosive gases is of vital significance [1,2,3]. Gas sensors are devices which are capable of achieving this requirement. Among these, resistive-type gas sensors based on metal-oxide-semiconductor (MOS) configurations are more attractive and widely used. The electrical resistance of the MOS gas sensing material is correspondingly changed with various types and concentrations of gases, which makes it convenient for gas testing. Sensitivity, selectivity, response and recovery times, and stability are the critical parameters of gas sensors. Considerable studies have been adopted to improve these gas sensing parameters by the modification of the nanostructures of the sensing materials, adding catalyst as well as synthesizing nanocomposites [4]. Because of easy fabrication, high sensitivity, and stability of MOS sensors under ambient atmosphere compared with other electronic devices, the correlative research on MOS-based gas sensors has become a hotspot in this area.

According to the different conductive behaviors, MOSs are classified mainly into two types, which are referred to as n-type and p-type. SnO_2_ [5] and ZnO [6] are the most representative MOS, and they both exhibit n-type oxide conductivity feature. Other n-type MOSs such as TiO_2_ [7], Fe_2_O_3_ [8], and In_2_O_3_ [9] are also widely studied by researchers for investigating their gas sensing behaviors. In contrast, p-type MOSs such as NiO, CuO, Co_3_O_4_, and Cr_2_O_3_ have received relatively less attention because of their lower response to target gases compared with n-type MOS. Some scholars such as M. Hübner et al. [10] suggested that with the identical morphological structures, the response of an n-type MOS-based gas sensor to target gases is equal to the square of that of a p-type MOS-based gas sensor. This indicates that the responses of p-type MOS-based sensors should be enhanced so as to ameliorate their gas sensing properties.

Nevertheless, regardless of whether MOS perform n-type or p-type conductive behavior, there exists the intractable problem of poor selectivity among some reduction gases due to their cross-sensitivity property, which makes it difficult to simultaneously quantify the concentration of target gases in the presence of interfering gases [11,12,13]. More attention should be paid that most p-type MOS exhibit excellent catalytic performance to promote selective oxidation of some volatile organic compounds (VOCs) [14,15]. Additionally, by compounding p-type and n-type MOS materials, p–n heterojunctions are created at the interface between these materials, whereby the transport behavior of their respective charge carriers will be changed. The combination of these two or more dissimilar components allows the integration of their different properties; therefore, it will enhance their comprehensive sensing performances and weaken their respective intrinsic sensing defects, so that the sensing parameters such as selectivity and sensitivity can be significantly improved.

The material structure incorporating two dissimilar components is often referred to as a heterostructure. The nanomaterials which constitute a heterostructure always have different Fermi levels. By creating electrical contact at the interface when contacting dissimilar semiconducting materials, the Fermi levels can equilibrate to the same energy, which results in a carrier transfer and the formation of a charge depletion layer [11,16]. This is the basic gas sensing enhancement mechanism of these heterostructures and will be discussed later.

**Figure 1:**
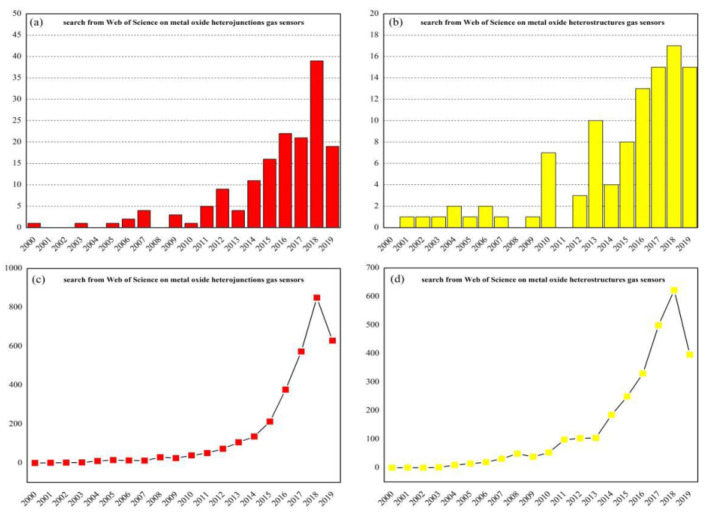
Records of the number of metal-oxide-heterojunctions-related published papers (**a**) or metal-oxide-heterostructures-related published papers (**b**) and the number of their respective times cited (**c**,**d**) since the year 2000. The search string: TITLE-ABS-KEY (metal and oxide and heterojunctions/heterostructures and gas and sensors) (internet search of Web of Science on 28 June 2019).

In recent years, there are several review articles which have been published about MOS nanomaterials applied in the gas sensing field. However, this review article specifically focuses on the MOS-heterostructured gas sensing materials [17,18]. Many researchers have found that the selectivity and other sensing parameters of resistive-type MOS gas sensors can be improved by synthesizing nanocomposites [19,20,21,22,23,24]. Attracted by the superior performances of gas sensors with heterostructures, researchers still maintain a high interest in nanostructured MOS which are used as based blocks to fabricate the heterostructures. Analyzing the data of the search from the Web of Science since the year 2000 (Figure 1), it can clearly be seen that the number of published papers on this topic is continually increasing. In addition, the number of times papers corresponding to heterojunctions or heterostructures gas sensors are cited is also gradually increasing.

## 2. Gas Sensing Mechanisms of Metal Oxide Semiconductors

**Figure 2:**
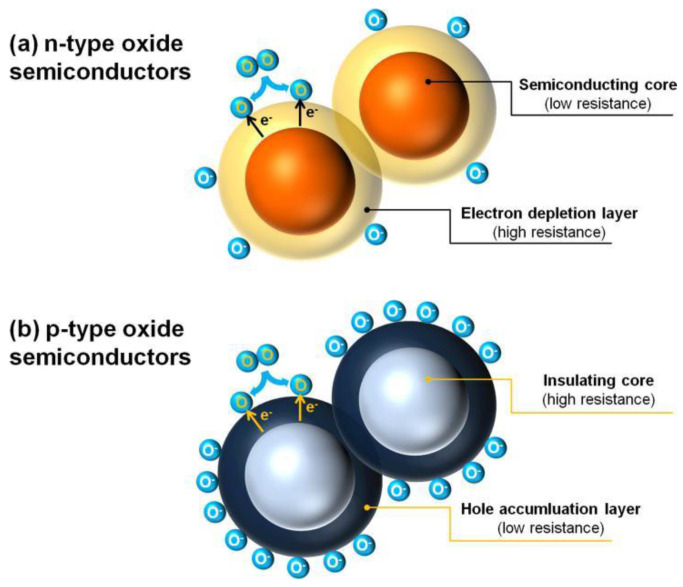
Formation of core–shell structures of charge carriers in (**a**) n-type and (**b**) p-type oxide semiconductors. Reproduced with permission from Ref. [25].

Electrons and holes are the main charge carriers of MOS for conductivity. According to the differences of their relative contents, n-type MOS are materials which carry more electrons than holes, while the relative content of internal carriers of p-type MOS is the opposite. When the electronic affinity of gas molecules is greater than the work function of MOS surface, some oxidizing gases (e.g., O_2_) are able to capture electrons from the MOS surface; thus, ionized oxygen anions (i.e., O_2_^−^, O^−^, and O^2−^) will form with the difference of temperature range. Furthermore, if the electronic affinity of gas molecules is less than the work function of MOS surface, electrons can release to the surface from the gas molecular, forming cations adsorption, such as reducing gases (CO and H_2_) substituting for oxidizing gases absorbed. Therefore, for an n-type MOS, when exposed to an oxidizing gas, electrons are captured and combined with the gas molecular, thus constructing an electron depletion layer on the surface of the material to establish electrical core–shell structure (Figure 2a), which exhibits a high resistance characteristic. When an n-type MOS is exposed to a reducing gas, adsorbed oxygen ions are able to be oxidized, the trapped electrons will re-inject into the MOS surface, thereby increasing the charge carrier concentration and obviously decreasing the sensor resistance. It is widely noted that there is a certain functional relationship between the response of MOS gas sensor and the gas concentration [12,25,26,27,28,29,30]. Thus, it will be helpful for gas quantitative analysis.

In contrast, by adsorbing oxygen, the charge carriers of p-type MOS are mainly holes which assemble on the surface of MOS to form a hole accumulation layer with low resistance; in contrast, the MOS performs a high resistance characteristic, which also establishes the core–shell configuration (Figure 2b). Under this configuration, when adsorbing reducing gases, the electrons are released to the MOS through the reaction between the reducing gases and the ionized oxygen anions on the surface of the materials, which, in turn, decreases the concentration of holes in the shell layer and increases the material resistance. Table 1 summarizes the converse gas sensing behaviors of n-type and p-type MOS materials.

On the basis of the aforementioned MOS gas sensing mechanisms, Yamazoe firstly proposed the grain-size effect which is illustrated as follows [31]. First, we introduce two concepts. One is ‘D’ which represents the grain size of the particles; the other is the Debye length (L_D_), which is defined as the furthest distance to which a fixed charge can provide a force to the surrounding charges. Additionally, L_D_ is approximately equal to the thickness of electron depletion layer or hole accumulation layer. The grain size mainly determines the response value of sensing materials if the grain size is greater than twice of the Debye length (D > 2L_D_), whereas it will bring some influence to the gas response of p-type MOS in which the conduction mostly occurs along the shell layer. Under this condition, the slight change of the concentration of holes in the shell layer because of the gain or loss of the electrons will not bring notable variation of the sensor resistance. This is the main reason why the response value of p-type semiconductor is lower than that of n-type semiconductor.

## 3. Heterostructure Classification

Before introducing the heterostructure synthesis methods and elucidating the sensing mechanisms of different heterostructural materials, it is worth describing the major classifications of heterostructural compound materials. The combination of these materials fabricates diverse heterojunctions, such as p–n, n–n, and p–p nanojunctions according to the semiconducting properties of sensing materials. The sensing mechanisms of these different heterojunctions will be separately described in the following parts.

The relationship between the structure and distribution state of the constituents can effectively influence the sensing performance of materials. Thus, it is worth describing three structure–architecture types of heterostructure by the following nomenclature.

- A dash between the names of two or more constituents such as SnO_2_-Co_3_O_4_ represents a simple mixture of SnO_2_ and Co_3_O_4_, which are not controlled and randomly distributed.
- An “@” sign between two or more constituents such as SnO_2_@CuO represents the base material SnO_2_ with a second material CuO adding on it in some ways. For example, CuO is coated on SnO_2_ in the ways such as sputtering, dipping, etc.
- A forward slash between constituents’ names such as ZnO/NiO represents a clear partition or a well-defined interface between these two materials. For example, ZnO/NiO could represent a bi-layer structure or core–shell ZnO/NiO nanorods.

### 3.1. Simple Mixed Compound Structures

This synthetic method is an easy way to obtain the mixed compound structure by mixing the existing oxide powders. B. Lyson-Sypien et al. [32] detected that by mechanical mixing with different contents (0%, 2%, 10%, 50%, and 80%) of TiO_2_ and SnO_2_, the sensing response to H_2_ gradually increases with the increase of TiO_2_ content and reaches the maximum with 50% TiO_2_, which is attributed to the presence of most heterojunctions in this composition. It should be noted that even though the nominal component of two heterostructural materials is identical, the gas sensing behavior can be quite different. The dispersion state of heterostructures, which depends on the processing routes, has a significant influence on the behavior of the sensor material. D. Shaposhnik et al. [33] studied the gas sensing behavior of TiO_2_-doped SnO_2_ by comparing co-precipitation with mechanical mixing method. The results showed that the optimum composition which performed the best sensitivity to H_2_ was 10 mol% TiO_2_ for co-precipitation and 20 mol% TiO_2_ for mechanical mixing.

### 3.2. Layered Structures

Heterostructures based on bi-layers or multi-layers exhibit well-defined interfaces, which are suitable for characterizing and modeling because of their simple stacked 2D structure. Thanks to this structure, it becomes easier to characterize their electronic properties at the interface. In addition, it is easy to study the thermal stability which includes the possible growth of mixed phases and the diffusion across the interface [34]. However, these structures are less popular for some applications due to the lower specific surface area ratio, which brings less gas-accessibility to the heterojunction interface, thus influencing the gas sensor response. Dandeneau et al. [35] optimized the porosity and crystallinity of the top CuO film by changing the pyrolysis temperature through the sol-gel process of n-ZnO/p-CuO heterojunctions so as to rapidly analyze the gas diffusion rate to the interface of heterojunctions.

### 3.3. Structures Decorated with Second-Phase Particles

Another common class of heterostructures is the decoration of the host oxide material with nanoparticles of a second phase, which includes oxide materials [36,37,38], metals [39,40] or carbon-based materials [41,42]. These structures are usually used in photocatalysis [43] and photovoltaics, of which the secondary particle phases can perform as catalysts or sensitizers so as to enhance the sensing performance. Moreover, noble metal nanoparticles such as Au, Ag, Pt, and Pd are also used as the second-phase particles which are added into host oxides [44,45,46,47] and which act as catalysts and activators to increase the dissociation and reaction rate of gas molecular by reducing the activation energy of the reactions [48]. However, these noble metal nanoparticles can increase the cost and instability issues, such as catalytic poisoning effect because of the activity decrease and phenomena of coarsening or clustering at high temperatures [49].

### 3.4. One-Dimensional Structures

These nanostructures include mainly nanowires, nanorods, and nanofibers, which usually possess large specific surface areas. The selectivity and gas sensing properties can be significantly improved by modifying the secondary-phase material on one-dimensional nanostructures, such as nanowires modified by nanocrystals [50,51,52], core–shell nanowires [53,54], core–shell nanotubes [55,56], and composite nanofibers [57,58,59,60,61]. Generally speaking, the gas sensing enhancement mechanisms attribute mainly to three aspects including one-dimensional structure model, heterojunction effect, and catalytic effect. One-dimensional heterostructure usually has a high length–diameter ratio, which means that more surface atoms can participate in the gas–solid reaction compared with other heterostructures [62]. The actual effective heterojunction area of these one-dimensional composites varies with the process types and synthetic methods, which will be introduced later.

### 3.5. Core–Shell Structures

**Figure 3:**
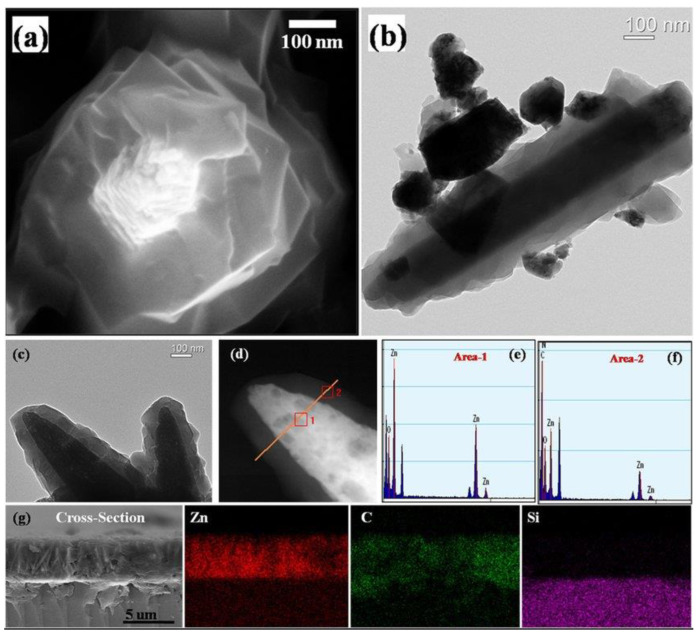
Microstructure of ZnO@ZIF-8 core–shell nanorod film: (**a**) SEM image; (**b**,**c**) TEM images; (**d**) HAADF-STEM image; (**e**,**f**) EDS element scanning of area-1 and area-2 in image (**d**); (**g**) cross-section image and EDXS mappings (reprinted with permission from Ref. [63]).

The last morphology to which we often pay attention is core–shell structures. Among all types of heterostructures for gas sensing applications, core–shell structures are the most promising types and will attract future researchers. This morphology can provide a maximized interfacial area with the help of completely covering the host material with a secondary phase while minimizing the amount of the material as bulk. Because of their unique structural features, core–shell structures integrate the properties of both internal and external materials and also compensate for their respective shortcomings. Scholars have conducted much research on core–shell structures recently. Wu et al. [63] found that the zeolitic imidazolate framework-8 (ZIF-8) shell, which is a stable metal–organic framework (MOF) porous material, had fine grains and was completely coated on the intact ZnO nanorod core, as shown in Figure 3a. The coating of the ZIF-8 shell was uniform and continuous, and the interfacial area between ZIF-8 shell and ZnO core was totally maximized, as shown in Figure 3b,c. It can be seen in Figure 3d–f that the core–shell structure of ZnO@ZIF-8 was particularly clear and the successful transition of ZnO core to ZIF-8 shell was found by EDS element scanning. The cross-section image and EDXS mappings of Figure 3g showed that ZIF-8 shell fully covered the ZnO nanorods, and the porous ZIF-8 shell could control access of the gas species to the ZnO core so as to improve the gas selectivity of the sensing materials.

## 4. Overview of Synthesis Methods

With the development of the research on metal-oxide-based heterostructures, different technologies have been employed to fabricate these materials. The preparation of these heterostructures demands various factors such as structural affection and chemical homogeneity, which lead to the rapid development of synthesis methods. This investigation will introduce some common fabrication techniques, making it possible to develop various heterostructural nanomaterials by combining different types of MOS materials.

### 4.1. Sol-Gel Method

**Figure 4:**
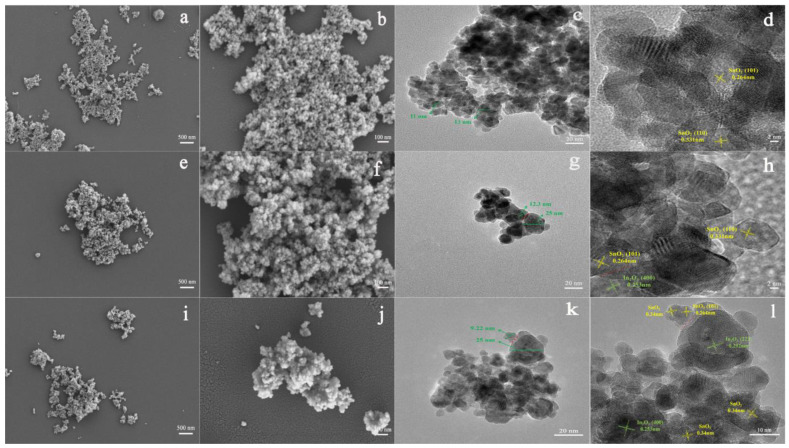
FESEM and TEM images of (**a**–**d**) pristine SnO_2_, (**e**–**h**) 20 mol%In_2_O_3_-SnO_2_ nanocomposite and (**i**–**l**) 20 mol%In_2_O_3_-Sn_0.92_In_0.08_O_2_ nanocomposite (reprinted with permission from Ref. [66]).

The sol-gel method has become one of the most preferable methods for fabricating MOS-based heterostructures. The compounds with high chemical activity act as precursors, which are evenly mixed in a liquid environment by adding surface active agent, forming a stable and transparent sol system by internal chemical reactions. The stagnant gel subsequently forms by slow polymerization. Finally, the nanostructured materials can be synthesized by drying and sintering methods. Jiang et al. studied the effect of polyethylene glycol on the microstructures of TiO_2_ thin films using the sol-gel method [64]. They reported that porous and fine-grained TiO_2_ films can form when adding more or with a high molecular weight of polyethylene glycol. They drew the conclusion that the sol-gel method could control the shape and size of fabrication materials by changing the solution composition and the synthesized conditions. In this aspect, many research groups synthesized different shapes of MOS heterostructures. Hernández et al. fabricated heterostructural materials based on TiO_2_ nanoparticles and ZnO nanowires by the sol-gel method [65]. Referring to our previous work, we successfully prepared pristine SnO_2_ (Figure 4a–d), SnO_2_-In_2_O_3_ heterostructure (Figure 4e–h), and In^3+^-doped SnO_2_-In_2_O_3_ heterostructure (Figure 4i–l) via the sol-gel method [66]. The structure morphology of the SnO_2_-In_2_O_3_ nanocomposite was not affected after In^3+^ doping modification. The particle size of SnO_2_ was changed by doping with In^3+^, which could improve sensing performance towards CO gas.

### 4.2. Hydrothermal–Solvothermal Synthesis Method

**Figure 5:**
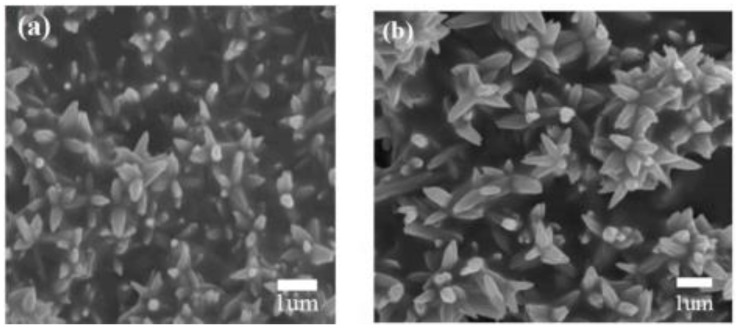
(**a**) FESEM image of pure ZnO nanoflowers; (**b**) FESEM image of NiO-decorated ZnO nanostructures (reprinted with permission from Ref. [68]).

Because of the morphology and structure of MOS-based heterostructures that play an important role in improving their gas sensing properties, many researchers have been making effort to fabricate heterostructures using novel methods. Among these, the hydrothermal–solvothermal synthesis method is considered to be a powerful and efficient route for the fabrication of diverse kinds of heterostructural semiconductor nanomaterials, which can precisely control the structure and morphology of MOS nanomaterials, achieving the goals of fabricating a wide spectrum of metal oxide heterojunctions. This method is performed in an autoclave, where the solution concentration, reaction time ,and temperature can be automatically controlled [67]. Therefore, phases with diverse morphology and properties are fabricated by controlling the reaction process and crystal growth. Due to allowing the fabrication of a wide spectrum of metal oxide heterostructures, this method is widely studied by most researchers. Recently, Liu et al. fabricated a flower-like structure composed of NiO-decorated ZnO nanostructures via a one-step hydrothermal procedure [68] (Figure 5). In addition, by varying the synthesis temperature and the solution concentration, a two-step hydrothermal method can construct heterostructures of the same materials with diverse shapes. Wang et al. studied the effect of hydrothermal temperature on the morphology of metal oxide heterostructures and successfully synthesized a nanobelt-like structure of SnO_2_-TiO_2_ at relatively high temperatures [69]. Liu et al. controlled the reaction time and temperature by means of solvothermal method and then obtained NiO/ZnO hollow spheres materials [70]. The aforementioned introductions illustrate that a precise control over the hydrothermal synthetic conditions is the key factor for the construction of high-quality metal oxide heterostructures with diverse shapes.

### 4.3. Vapor Deposition Method

This method mainly consists of chemical vapor deposition (CVD) or physical vapor deposition (PVD). CVD technology uses mainly one or several gaseous compounds or elementary substances containing film elements to produce films by chemical reaction on the substrate surface. CVD method can be used to purify materials, fabricate new crystals, as well as deposit monocrystal, polycrystal, glassy inorganic films, etc. The physical properties of materials can be precisely controlled by the process of vapor deposition. Additionally, high purity samples with different structure and morphology can be obtained by CVD method through precise control of vapor deposition process, such as the operating temperature, the pressure in the reactor, the template material, as well as the composition of the gas-phase [71]. Recently, by preparing SnO_2_/ZnO superlattice nanowires, Jiang et al. found that Au loading is helpful for the adsorption of Zn/Sn vapor and the formation of SnO_2_ superlattice on the ZnO lattice surface [72].

Another well-developed technique to synthesize novel heterostructures is PVD. Different catalyst layers could be deposited on the substrates to improve the nucleation of oxide materials. The catalyst type, the patterned template, the temperature and pressure inside the furnace, and the carrier gas composition and its flow affect the morphology of materials prepared by PVD method [73]. The PVD method is often carried out at high temperatures in a high-vacuum or inert-gas environment, which obtains low dimensional metal oxide heterostructures. Choi et al. deposited a thin Au layer on the surface of ZnO nanofiber stems to promote the growth of SnO_2_ nanowires by PVD method and successfully fabricated the ZnO-SnO_2_ nanofiber–nanowire stem–branch heterostructure [74].

### 4.4. Electrospinning Method

**Figure 6:**
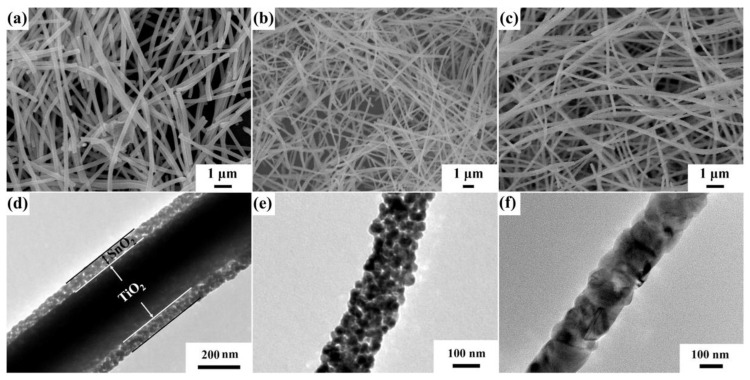
Field emission scanning electron microscopy (FESEM) images of (**a**) TiO_2_-SnO_2_ core–shell nanofibers (NFs), (**b**) SnO_2_ NFs, and (**c**) TiO_2_ core–shell nanofibers, transmission electron microscopy (TEM) images of (**d**) TiO_2_-SnO_2_ core–shell NFs, (**e**) SnO_2_ NFs, and (**f**) TiO_2_ core–shell NFs (reprinted with permission from Ref. [76]).

Electrospinning is a technique which utilizes high voltage electrostatic field force to fabricate nanofibers. In this way, the polymer and related materials can be prepared into one-dimensional nanofibers with high specific surface area, controllable composition and shape, and porous structure after calcination. The working mechanism of this technology is that the polymer or solution is electrified with the help of a high-voltage electrostatic field, and the liquid drop at the tip of the nozzle will form a suspended liquid drop (i.e., “Taylor cone”) and extend from the tip of the cone to obtain the fiber filament. Meanwhile, the liquid drops are subject to surface tension and surface charge repulsion force caused by the electrostatic field. When the surface charge repulsion force is greater than the surface tension, the micro flow of polymer will be ejected from the solution surface. The liquid flow is stretched and dragged by electric field force, then the solvent volatilizes and solidifies, and finally the sample is deposited on the prepared substrate to form polymer fiber [75]. Nanopolymer filaments can be fabricated by electrospinning. Feng et al. [76] prepared TiO_2_-SnO_2_ composite heterojunction materials with core–shell nanofiber structure by the electrospinning method (Figure 6).

## 5. Mechanisms of Gas Sensing Enhancement with Heterostructures

### 5.1. Working Mechanisms of Gas Sensing Materials

The sensing mechanism of MOS materials consists mainly in the interactions of the target gas molecules with the pre-absorbed oxygen species on the surface of gas sensing materials [77,78]. Referring to a resistive-type metal oxide gas sensor, the signal transmission mechanism is dependent on the change of the electrical resistance or conductance of sensitive materials when interacting with the analytic gas. The resistance of MOS gas sensors may increase or decrease on exposure to the gas depending on the type of metal oxide and gas analyte [79,80,81]. Different semiconducting materials can form various heterojunctions, which, in turn, integrate their respective conduction properties. Compared with single gas sensing materials, the construction of heterostructured compound materials can improve the sensing performance of gas sensors. Therefore, to understand the gas sensing mechanisms of materials composed of heterojunctions, exploring the junction sensing behavior is of vital importance.

### 5.2. Role of Heterojunction at the Interface

Two different solid-state materials can construct an electronic junction at the interface between them, which is always called a heterojunction. The study on heterojunction interface is significant when analyzing the mechanism of composite semiconducting materials. The p–n junctions are the most common heterojunctions used to modulate gas sensing properties.

Referring to the heterostructures gas sensing literature, n-type MOS gas sensors are more attractive to researchers in comparison with p-type MOS gas sensors because of several considerations such as better stability and higher sensitivity [82,83]. The combination of these two types of sensing materials opens a novel way to better improve the comprehensive sensing performance of gas sensors. In addition, these materials can be constructed into different structure–architecture types of heterostructure [84,85,86,87]. For reference, some sensing materials with their respective conduction types are listed in Table 2.

Because of the difference in the Fermi energy (E_F_) between two different sensing materials—for example, the E_F_ of n-type semiconductor material is always higher than that of the p-type semiconductor material when forming heterostructure—if they connect to form a heterostructure, the electrons at the higher energies will flow across the interface to unoccupied lower energies states until E_F_ reach equilibrium, which results in the recombination between electron and hole in the vicinity of a p–n junction. This decreases the concentration of charge carriers and leads to the creation of a charge carrier depleted zone at the interface called the depletion region. This phenomenon is often called “Fermi level-mediated charge transfer”. Because of the band bending, a potential energy barrier will develop at the interface, which is caused by the difference in the original E_F_ of the materials. Therefore, in order to pass through the interface, charge carriers must overcome this potential energy barrier. According to the aforementioned conduction types of sensing materials, the mechanisms of p–n, n–n, and p–p heterojunctions on gas sensing will be discussed in the following sections.

#### 5.2.1. p–n Nanojunctions

**Figure 7:**
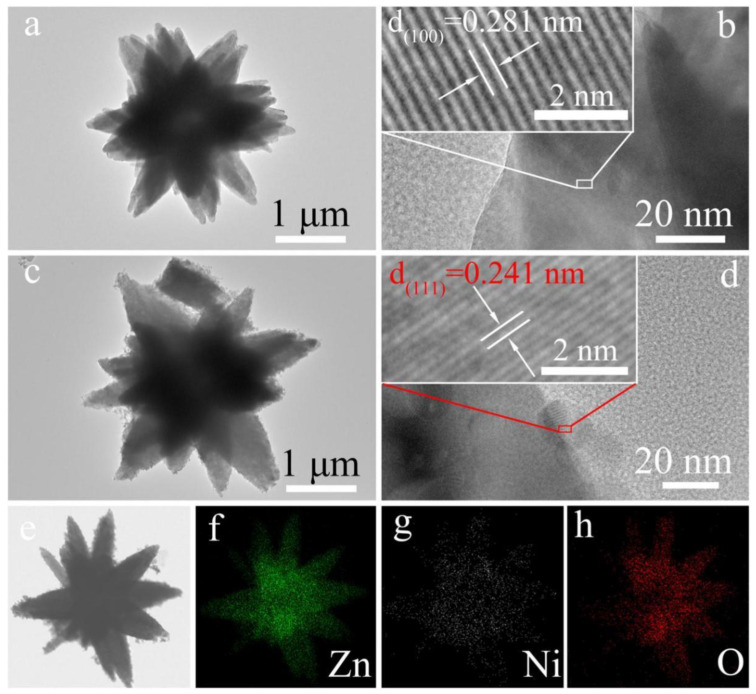
(**a**,**b**) TEM image and corresponding HRTEM image of a single pure ZnO microflower; (**c**,**d**) TEM image and corresponding HRTEM image of NiO-decorated ZnO to form NiO-ZnO composite microflower; and (**e**–**h**) The corresponding EDS elemental mapping images. (Reprinted with permission from Ref. [89]).

Considering a flower-like n-type ZnO decorated with p-type NiO nanoparticles (as shown in Figure 7) [89], the normal ambient resistance of NiO-decorated ZnO flower-like heterojunctions in air is higher than that of single pure ZnO microflowers. However, when acetone is introduced, a large decrease of the resistance (R_g_) may occur, resulting in a large change of gas response for sensing materials; this is because the initial value of Ra is exceedingly high, as the gas response is defined as R_a_/R_g_ for reducing gas [90]. Therefore, the response to target gas is obviously increased. The likely reasons can be interpreted as follows. First, increasing the concentration of initial absorbed oxygen ions helps to improve the gas sensitivity. NiO generally exhibits better oxygen-adsorption ability. With the help of the catalytic function of NiO, oxygen ions can be easily absorbed on the surface of NiO, and Ni^2+^ can be oxidized to a higher oxidation state (Ni^3+^), which significantly enhances the concentration of surface adsorbed oxygen. Secondly, the conduction of charge carriers across the heterojunction interface can contribute to the gas sensing performance. Due to the formation of an energy barrier potential at the interface between n-type ZnO and p-type NiO, the conduction capability of electrons and holes across the p–n interface will be weakened. When absorbing acetone, the height of energy barrier potential efficiently decreases, contributing to the conduction of charge carriers and increasing the sensing response.

**Figure 8:**
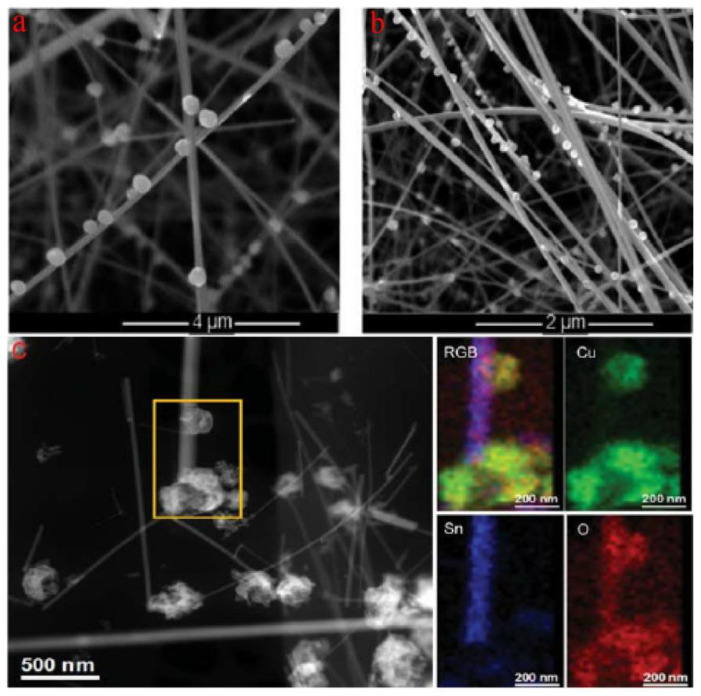
(**a**,**b**) SEM micrographs of CuO particle-decorated SnO_2_ nanowires deposited on Al_2_O_3_ substrate; (**c**) STEM image of SnO_2_@CuO heterostructures (left) and EDS elemental maps (right) (reprinted with permission from Ref. [91]).

**Figure 9:**
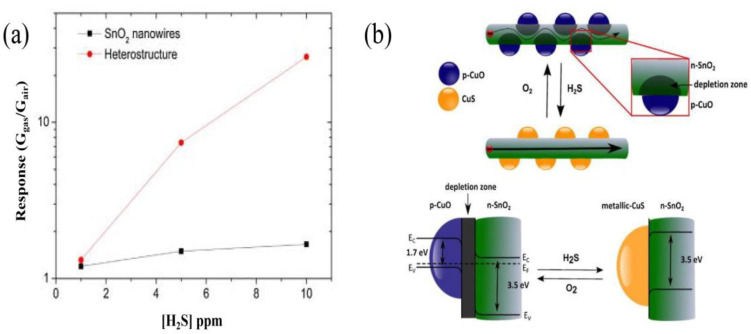
(**a**) A significant improvement in sensor response to H_2_S is observed when forming p–n junctions. (**b**) Sensing mechanism of CuO sensitivity to H_2_S is explained. At the interface between SnO_2_ and CuO, a depletion zone forms in air. After H_2_S joins in, p-CuO particles react with it and transforms to CuS, resulting in decreasing the depletion region. (Reprinted with permission from Ref. [92]).

Irina et al. reported the synthesis of p-type CuO nanoparticles decorating SnO_2_ nanowires, constructing SnO_2_@CuO nanowires by chemical vapor deposition (CVD) for enhancing the detection of H_2_S [91]. Figure 8a,b show the successful synthesis of SnO_2_ nanowires decorated with CuO nanoparticles. It is also shown in Figure 8c that the successful formation of CuO-SnO_2_ heterostructure by STEM and EDS element analysis. This research has certified that the CuO decorating narrows the conduction channel of SnO_2_ when forming CuO-SnO_2_ heterostructures, increasing the initial resistance of sensors in air. Quite interestingly, when absorbing H_2_S, p-type CuO will react with H_2_S and be sulfuretted into CuS, destroying the potential energy barrier at the interface. Additionally, the hydrogen ions will transfer on the surface of the host material, reacting with the adsorbed oxygen ions and, thus, also decreasing the electrical resistance [92]. These are all attributed to the strong chemical interaction between H_2_S and CuO, as shown in Figure 9b. Irina et al. also performed a gas sensitivity test. It can be seen in Figure 9a that the response of CuO-SnO_2_ heterostructure is obviously higher than that of pristine SnO_2_ nanowires, certifying the sensing enhancement in heterostructures. The same phenomenon was also reported in the form of CuO-ZnO heterostructures for H_2_S testing. The potential energy barrier height of heterojunctions was efficiently reduced because of the chemical interaction between H_2_S and CuO [93].

It is worth noting that Mashock et al. reported an opposite heterostructure configuration of SnO_2_ nanoparticles coating CuO nanowires [94]. Similarly, an energy barrier potential forms at the interface between n-type SnO_2_ and p-type CuO. Since p-type CuO is dominated by surface conduction because of the accumulation layer formed in air, the effect of SnO_2_ deposition is worth considering. The study results showed that with the deposition of SnO_2_ nanoparticles coating, a significant increase in the electrical resistance occurred when forming CuO-SnO_2_ heterojunctions. Furthermore, by doubling the deposition time of SnO_2_, they could create a continuous coating and a core–shell structure was successfully constructed. Prolonging the deposition times contributed to bringing an even higher resistance, which also produced a smaller increase in response to NH_3_ compared with the nanowires of shorter deposition time. The authors came up with two possible mechanisms to explain this phenomenon: (1) NH_3_ lowers the hole concentration of CuO nanowire by electron transfer, which increases the resistance; and/or (2) the increase of electron concentration in SnO_2_ nanoparticle due to removing absorbed oxygen enhances the p–n junction. This strong nanojunction is able to block the hole transport and increase the resistance.

#### 5.2.2. n–n and p–p Nanojunctions

Just like p–n heterojunctions, band bending of energy levels can also appear in n–n and p–p heterojunctions [95,96,97,98,99,100]. Among these reports, n–n heterostructures are widely studied by researchers for gas sensing. Different from a p–n junction with fewer electrons at the interface due to electron-hole recombination that increases the resistance, electrons are not exhausted in an n–n junction, and electrons from the material with a high Fermi level can easily transfer to the one with a low Fermi level, which then forms an accumulation layer rather than a depletion layer. While this accumulation layer will be depleted by the subsequent oxygen adsorption on the surface, further increasing the potential energy barrier at the interface to enhance the sensing performance.

**Figure 10:**
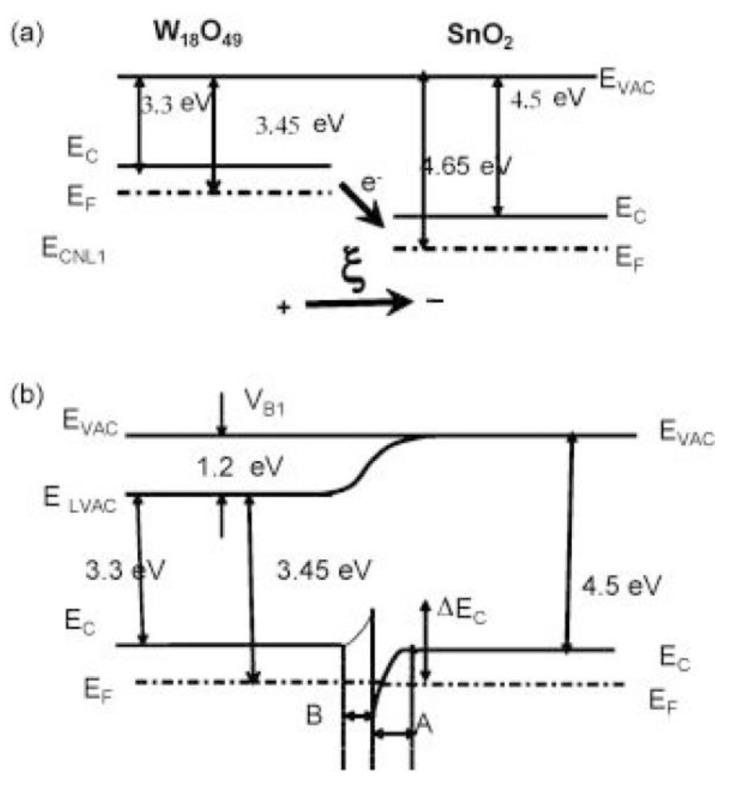
The energy band diagram of the gas sensing mechanism for (**a**) W_18_O_49_ and SnO_2_ materials and (**b**) the SnO_2_/W_18_O_49_ nanostructures. Reprinted with permission from Ref. [95].

Considering small n-type W_18_O_49_ nanowires grown on larger n-type SnO_2_ nanowires (Figure 10) [95], the energy conduction band of SnO_2_ is higher than that of W_18_O_49_. When they reach an equilibrium state with each other, electrons will transfer from the high energy conduction band of SnO_2_ to W_18_O_49_ until their Fermi energy levels achieve equilibrium, leading to the band bending at the interface between them. When exposed to the air, oxygen species can adsorb on the surface of SnO_2_/W_18_O_49_ nano-heterostructures. By capturing free electrons on the surface of heterostructures, these oxygen species can be ionized into oxygen ions, leading to the formation of an electron depletion layer, increasing the energy barrier height at the interface, which improves the H_2_S sensing performance.

**Figure 11:**
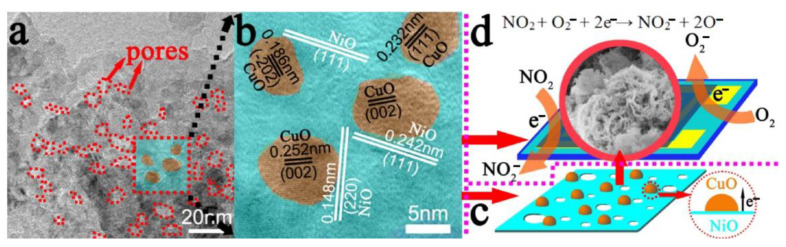
(**a**) TEM image of mesoporous NiO@CuO nanosheets; (**b**,**c**) heterojunction between NiO nanosheets and CuO nanoparticles at the interface; (**d**) sensing mechanism of NiO@CuO gas sensors exposed to air and NO_2_. Reprinted with permission from Ref. [98].

On the other hand, when referring to p-p heterostructure gas sensors, there are rare reports in the literature since the year 2000. Quite interestingly, recently in 2017, a paper was published on highly sensitive NO_2_ sensors based on NiO@CuO nanocomposite worked at room temperature [98]. The increased gas sensing property towards the detection of NO_2_ may be caused by two factors. Firstly, mesoporous hierarchical flower-like NiO nanosheet owns a large surface area, which performs as the catalyst and allows NO_2_ to adsorb and desorb effectively on the surface of the pores. Secondly, p-type CuO and p-type NiO are able to form the p–p heterojunction which plays a vital role in improving the sensing performance (Figure 11a–c). It helps to efficiently transfer the electrons from NiO to CuO. When the Fermi energy arrives at equilibrium state, the hole accumulation layer will form at the interface between NiO and CuO. O_2_ molecules will act as electron acceptors and capture electrons from the conduction band of the sensing materials by being absorbed on the surface of the sensing materials in air, which increases the holes concentration and decreases the resistance of the sensors. If these sensors are exposed to NO_2_, the NO_2_ molecules could interact with the chemisorbed oxygen ions on the surface of the materials and directly be adsorbed on the surface, which can decrease the resistance under NO_2_ atmosphere (Figure 11d). It may provide a novel method to detect NO_2_ gas at room temperature by constructing NiO@CuO heterostructure gas sensors.

### 5.3. Synergistic Effect

An additional mechanism that should be considered to enhance gas sensing performance is synergistic behavior which occurs in the heterostructured composite materials. These materials synthesize the advantages of each component, which results in a special synergistic effect between each components to improve the gas sensing performance of the materials. Generally speaking, it is when two different components of a material respectively contact with the gas phase and each provides a different purpose which is complementary to the other [11]. Ivanovskaya et al. found that composite oxide materials which simultaneously have acidic and alkaline active sites could decompose the organic gas molecules more completely due to the diverse redox properties of these composite oxide materials [101]. Costello et al. found that SnO_2_ could completely oxidize butanol to butyral and that ZnO had no effect on butanol decomposition while it could easily decompose butyral; moreover, ZnO-SnO_2_ composite oxides could synergistically decompose butanol and performed the highest sensing response [102]. Recently, Kamble et al. proposed that the synergism of Cr and noble metal Pt-activated SnO_2_ gas sensors could exhibit enhanced sensitivity and improved selectivity toward CO gas, which, respectively, synthesize the advantages of Cr for improving selectivity and Pt for enhancing sensitivity [103].

### 5.4. Catalyzed Spill-Over Effect

The catalyzed spill-over effect is another important sensing behavior often mentioned in the literature [29,104,105,106,107]. In general, the target gas molecules firstly react with one of the heterostructure composite constituents, forming a secondary product which remains adsorbed on the surface of the other constituent and directly affects the sensing properties. This phenomenon often occurs in CuO composite materials for H_2_S detection [91,92,108,109]. As aforementioned, with regard to CuO/SnO_2_ nanojunction composites, H_2_S can react with CuO nanoparticles and transform them into CuS. Then the left-over hydrogen spills over on the surface of the composite material, acting as a reducing agent and reacting with the other host material, thus decreasing the resistance [91]. Meanwhile, CuO plays a part in improving the sensitivity of host materials to H_2_S. Moreover, the spill-over effect can improve the sensing response by removing the depletion layer. Shao et al. [91] discovered that CuO was firstly combined with SnO_2_ to form a p–n heterojunction, which increased the resistance in air. However, when H_2_S was introduced in this p–n junction, CuO was converted to CuS and this new product acted as a moderate conductor between CuO and SnO_2_, When the conversion was completed, an ohmic junction could form between CuS and SnO_2_, eliminating the depletion region barrier of CuO/SnO_2_, thus favoring the conduction across the interface.

In addition, the spill-over effect applies to noble metals which are used to catalyze MOS materials. For example, noble metal Pt performs a strong catalytic effect at the surface of SnO_2_ [110]. It can catalyze the dissociation of O_2_ and spill over the oxygen ions which adsorbed on the surface of SnO_2_. The main advantage of Pt is to absorb exceptionally large number of gas molecules and convert them into adsorbed ions, decreasing the activation energy of MOS needed for reaction, hence reducing the response and recovery time and lowering the operating temperature [88].

### 5.5. Response Inversion Effect

**Figure 12:**
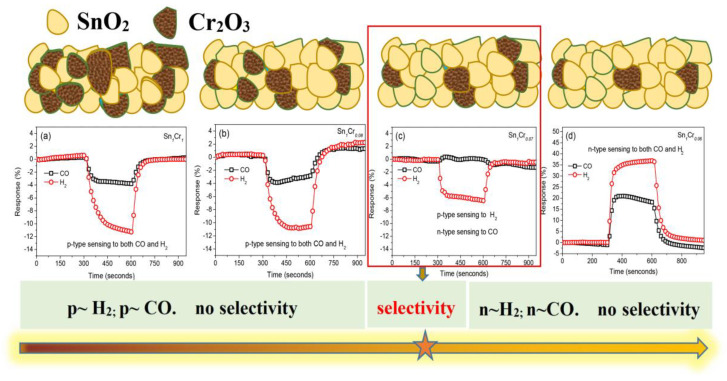
(**a**–**d**) Gas sensing response of SnO_2_-Cr_2_O_3_ heterostructure nanocomposite towards CO and H_2_ via changing Cr_2_O_3_ content. Reprinted with permission from Ref. [114].

The addition of a p-type material to an n-type material can form an n–p composite oxide material. It should be realized that under certain conditions, the p-type constituent can counteract the resistance change of the n-type constituent to target gases. Sometimes the p-type constituent can dominate the sensing properties over a certain range of constitution, making the composite oxide material present a p-type response. Several studies reported the n–p or n–p–n response inversion phenomena [111,112]. Kosc et al. [111] studied a bi-phase TiO_2_/NiO sputtered film which showed p-type conductivity. When absorbing a certain concentration of H_2_, the holes in the NiO layer would be fully compensated by the added electrons from broken oxygen bonds, resulting in the response type inversion, then the sensor presented n-type behavior. Additionally, Huang et al. [112] synthesized the ZnO-modified SnO_2_ nanorods, which showed a typical n–p–n response inversion to H_2_. Furthermore, it is worth noting that the response inversion phenomenon also occurs in p–n heterostructure nanocomposites when exposed to homogeneous gases by adjusting the metal oxide ratio. Yin’s research group has overcome the drawback of poor selectivity of MOS-based gas sensors to homogeneous gases such as CO and H_2_ [113,114]. As exhibited in Figure 12, due to the different adsorption tendency of CO and H_2_ on the surface of SnO_2_ and Cr_2_O_3_, under the optimal heterojunction composition (Figure 12c), H_2_ molecules prefer to adsorb on Cr_2_O_3_, causing the reduction of hole accumulation layer (HAL) on Cr_2_O_3_, which is greater than the decrease of electron depletion layer (EDL) on SnO_2_, and the increase of electron content is lower than the decrease of hole content. The hole content variation dominates the conductivity of n-SnO_2_-p-Cr_2_O_3_ heterostructure, while CO gas prefers to adsorb on SnO_2_, which brings about the opposite change tendency of conductivity; thus, sensing behavior under this proportion presents a p-type response and n-type response toward H_2_ and CO, respectively, successfully distinguishing these two homogeneous gases.

## 6. Conclusions and Future Outlook

The present work reported here has started to reveal novel and innovative ways to synthesize MOS heterostructural materials for improving chemical gas sensor performance. In this paper, a series of works by researchers in this field have been summarized. It is demonstrated throughout this review that it is possible to utilize different synthesis methods for preparing metal oxide heterostructures with different morphologies and shapes, each of them exhibiting a specific sensing performance towards particular target gas.

This literature review summarizes four different techniques which have been widely adopted for fabricating metal oxide heterostructures, namely core–shell, bi-layer, hollow spheres, and branched ones. The aforementioned methods have some advantages, such as the obvious heterostructure interface between different phases, the selective growth on the substrates, and the fabrication of multi-layered structures or composites. In recent years, more attention has been paid to the hydrothermal method due to the development of MOS materials with diverse morphologies and structures, such as nanorod-like, nanoflower-like, nanobelt-like, or hollow spheres structures by precisely controlling the reaction process. In addition, it seems that the combination of different fabrication methods is a more efficient way to acquire metal oxide heterostructures with novel morphologies or dimensions (core–shell nanostructures, one-dimensional heterostructures, two-dimensional layered heterostructures, or three-dimensional hierarchical heterostructures).

Furthermore, gas sensing enhancement mechanisms of MOS-based heterostructure materials have been demonstrated, which will make it useful to fabricate the corresponding heterostructure toward a specific target gas. However, despite novel and advanced technologies in the MOS field, nanotechnology and these complex configurations are now facing new challenges and issues, such as long-term stability, reproducibility, gas selectivity, characterization of the key parameters, establishing the gas sensing mechanism, and functional integration, which should be overcome in time. Recent works have provided us with new ideas for fabricating metal oxide heterostructures as gas sensing materials. Going forward, the synthesis of novel materials and the design of heterostructures will require further study of the influencing factors and the gas sensing enhancement mechanisms. Further study on MOS heterostructural semiconductor gas sensors should focus mainly on (1) novel materials and new heterojunction interfaces, and (2) the mechanisms which contribute to the gas sensing performance.

Although the present research focuses mostly on the combination of binary compounds, it should be realized that the interface of heterojunction is not limited to binary compounds, as there are many other configurations which could be used to prepare heterojunctions. In particular, composite materials obtained by the combination of metal oxides have proved to be extremely interesting for gas sensing applications. In addition, further research on heterojunction should not be limited to metal oxide, as organic and hybrid organic/inorganic composite materials also show great potential as gas sensors. Core–shell structure has much potential due to the maximization of the interfacial heterojunction area, where the electronic interaction is the most dominant. Self-assembly of 0D, 1D, and 2D structures can form unique morphologies and shapes. At present, it is still necessary to develop new synthesis methods to self-assemble these types of structures to fabricate special heterostructures.

Moreover, there are different opinions on the gas sensing mechanism, among which the most prominent ones are the grain boundary–barrier model, heterojunction structure, surface synergistic effect, catalyzed spill-over effect, response inversion effect, and separation of charge carriers effect. It is possible to design complex structures with the rapid development of new fabrication techniques. Therefore, it becomes essential to clarify the gas sensing mechanisms in order to properly select the morphology and heterostructure for a given application. On the basis of the first principles, the construction of gas adsorption model of metal oxide heterojunction is expected to clarify the gas sensing mechanism qualitatively.

## Figures and Tables

**Table 1:** Gas sensing behaviors of n-type and p-type materials to reducing and oxidizing gases.

Material Type	Dominant Charge Carrier	Reducing Gas	Oxidizing Gas
n-type	electrons (e^−^)	resistance decreases	resistance increases
p-type	holes (h^+^)	resistance increases	resistance decreases

**Table 2:** Conduction types of MOS and semiconductors (adapted from Ref. [88]).

Material Type of Conductivity	Materials	
	MOS	Semiconductors
n	SnO_2_, ZnO, TiO_2_, Al_2_O_3_, In_2_O_3_, V_2_O_5_, WO_3_	SiC, g-C_3_N_4_, GaN
p	CuO, NiO, Co_3_O_4_, PdO, Cr_2_O_3_, Y_2_O_3_	
n, p	Fe_2_O_3_, HgO_2_	Si, GaAs, InP

## Data Availability

Not applicable.
